# Dr. Aberdour, on Vaccination

**Published:** 1804-02-01

**Authors:** Alex. Aberdour

**Affiliations:** Prince's Place, Lambeth


### *To the Editors of the Medical and Physical Journal.*

GENTLEMEN,

AT a time like the present, when the small-pox are so prevalent, the illustration of any cases, which may tend to avert or lessen an impending evil, appears to me important.

With this view I beg leave to lay the following before the public:

About a twelvemonth ago, Diana Deemar, a girl four years old, caught the infection of the small-pox. Her mother brought her to me, with her sister, Elizabeth Deemar, a child about eighteen months old, to the New Finsbury Dispensary, to which institution I was then physician.

Both children slept together in the same bed.

The variolous eruption in Diana Deemar was at the height.

Elizabeth Deemar had a puny appearance, laboured at the time under the hooping cough, and suffered much from dentition.

Notwithstanding these disadvantages and the little prospect of success, added to the risque of an imputation of blame being attached to my professional character, I recommended to the mother to have the child inoculated for the cow-pox. It was done.

In the course of the first week the inoculated arm had a promising appearance, and I was in great hopes of success. In this, however, I was disappointed, the part inoculated had inflamed, but did not exhibit the genuine characteristics of the vaccine pock, and by the tenth day the inflammation was almost gone. I would then have repeated the inoculation, but did not for want of vaccine matter.

On the 16th day from the inoculation, I procured some recent matter from the Bloomsbury Dispensary, with which I inoculated my little patient the second time. No symptoms of small-pox had hitherto appeared. In less than four days from the second inoculation, the genuine vaccine pock appeared on the arm where the first inoculation had been performed, and presented the appearance of what is usually observed on the eighth day of inoculation.

Shortly afterwards there appeared about seven pustules,

 the the greater number of which were on the face; all these died away within three days.

I would only observe, that in the above case there can be little doubt of the variolous contagion having entered the system, as appears from the spurious pustules, which subsequently shewed themselves; this was counteracted and rendered in a great measure effete by the first inoculation. It seemed to require, however, a second inoculation to insure the victory of the vaccine pock.

The sequel of the case is, the child soon recovered from the hooping cough, and every distressing symptom arising from dentition; and has in the course of the last twelvemonths enjoyed a much better state of health than she did prior to the inoculation.

The father's name is Joseph Deemar, who lives with his family at No. 11, Green Street, Theobald's Road.

On the fifth instant, I was desired to see a child named Elizabeth Roffy, nearly six months old, who was ill of the small pox, and the seventh day of the eruption. The child was suckled by a Mrs. Cave, who also gave suck to her own child, Mary Ann Cave, a girl about a month older than the other. Mrs. C. had also a son named John Cave, two years old.

I recommended to the mother to have her own children inoculated for the cow-pox, which was done the following day, the sixth instant. In a few days the usual marks of infection having taken place, appeared in the boy, and went through the common course. On the 16th, however, several pustules shewed themselves on different parts to the number from twelve to eighteen, all which died away in a few days, and never put on a purulent form.

In the girl, the inoculated parts seemed to be not a little inflamed the first five days, but disappeared on the seventh day of the inoculation, viz. the twelfth instant. On this day, the inoculation was repeated.

On the thirteenth, an eruption appeared on the face and different parts of the body, which left no doubt of its being the small pox, and which turned out to be of the confluent kind.

In this case the attempt to dislodge the enemy failed. I am of opinion, however, that the virus of the small pox, previously introduced into the system, was somewhat meliorated by the vaccine inoculation, and I entertain the hope that the child will utimately do well, at least this appears appears to me to be the case on the day in which I now write, viz. the 22d instant.

On the 6th instant, Catherine Bold, a child four years old, was seized with the confluent small-pox of a very bad kind, and died on Friday the 16th instant.

The mother brought her brother, Edmund Bold, a child two years old, to the station of the Jennerian Society, in Castle Street, King's Mews, to be inoculated for the cow-pox, which was performed by Mr. Cullrune, of Pimlico, on the 12th instant.

I saw the child with Mr. Cullrune on the 19th. The inoculated arm had the usual appearance. A few days before, seven or eight pustules appeared on different parts of the body, but these had died away by this time.

The father, William Bold, a journeyman tradesman, lives at No. 7, Old Round Court, in the Strand.

On the 24th ult. Elizabeth House was seized with the small pox, which made their appearance on the same day. She died on the 4th instant.

Mr. Diamond, of Holborn, who had been consulted on the occasion, recommended to the mother to have her little boy, about eighteen months old, inoculated for the cow-pox. This was performed, I believe, by Mr. Waschel, of the Small-Pox Hospital, on the 2d. instant.

The inoculated arm discovered the usual appearances of the infection having succeeded.

On the 11th instant, the small-pox made their appearance, which were very distinct and rather numerous; these turned however on the seventh day, and the child underwent a mild disease.

On this case I would observe, that both the infections seemed to have contended for victory, and between them it may be said to have been a drawn battle.

To the above cases, I beg leave to subjoin one of the casual cow pox; it is meant as an answer to the few sceptics who still doubt the efficacy of the vaccine pock, and by whom I have heard it stated that the cow-pox was only a security against the small-pox for a few years.

It is needless to add that the objection was made without any foundation; and I trust that, ere long, such opinions, like the clouds vanishing before the splendor of a meridian sun, will be annihilated before the cause of truth.

About fifty years ago, a Mr. Packer was a servant to a farmer in Brydon Forest. He was then employed daily in milking ten or twelve cows. In this business he was affected with the cow-pox in one of his hands, and underwent rather a severe disease.

Sometime after he went and lived as a servant with a Mr. Byam, surgeon, in Cirencester.

Mr. Byam going to inoculate his own child for the small pox, proposed that Packer should also undergo the operation, which was done, but he did not take the disease. He was afterwards exposed on different occasions to the variolous infection, but was never affected by it. He was much at a loss to account for it until within these few years.

The old gentleman now lives at No. 3, North Street, John Street, Tottenham-Court Road.

I am, &c.

*Prince's Place, Lambeth,*
*December* 22, 1803.

ALEX. ABERDOUR.

P. S. The prognostic which I gave respecting the child Mary Ann Cave, has been verified. The pustules contained a reddish kind of serum, and none of the brown yellow purulent matter of the small-pox. Their figure was a medium between the flattened surface of the vaccine pock and the conical apex of the distinct small-pox.

The secondary fever ushered in by a few short rigors, was of short duration; the fœtor, the tumefaction of the face, and the salivation, which attend the confluent small-pox, were wanting in this case.

The common saline mixture with small doses of antimonials, gentle laxatives, an attention to cleanliness, and the antiphlogistic regimen, constituted the whole of the treatment. Blisters and opiates were unnecessary. The breast supplied the child with her only food.

Out of several hundred cases of confluent small-pox, which I have in the course of practice had occasion to see, this case is without exception the mildest confluent disease I have ever witnessed.

From the cases above related, I am decidedly of opinion, that practitioners have it in their power to arrest the progress of small-pox, although the disease may have entered into a family. The chief obstacles are the prejudices which still exist among the lower classes of the community.

A CASE

